# Caffeine Intake During Pregnancy and Neonatal Anthropometric Parameters

**DOI:** 10.3390/nu11040806

**Published:** 2019-04-09

**Authors:** Regina Wierzejska, Mirosław Jarosz, Barbara Wojda

**Affiliations:** Department of Nutrition and Dietetics, Clinic of Metabolic Diseases and Gastroenterology, Institute of Food and Nutrition, 02-903 Warsaw, Poland; jarosz.zaklad@izz.waw.pl (M.J.); bwojda@izz.waw.pl (B.W.)

**Keywords:** caffeine, coffee, tea, energy drinks, pregnancy, newborn

## Abstract

Caffeine is a psychoactive substance that may affect the normal course of pregnancy, therefore its intake during that time should not exceed 200 mg/day. The aim of this study was to evaluate caffeine intake among pregnant women from the Warsaw region. The study was conducted among 100 pregnant women who delivered at the Department of Obstetrics, Gynecology and Oncology, Medical University of Warsaw. Caffeine intake from coffee, tea, and energy drinks was measured using a questionnaire. Direct interviewing was used, with all interviews conducted by the same dietitian. Multiple regression analysis was used to investigate the relationship between caffeine intake and anthropometric measurements of the newborns. Mean caffeine intake among pregnant women was 68 ± 51 mg/day. Only 2% of the respondents exceeded the safe dose of 200 mg. Tea (mostly black) was the source of 63% of all caffeine. No relationships were found between caffeine intake and neonatal weight, length, or head and chest circumference (*p* > 0.05). Caffeine intake in our study population was relatively low and did not negatively affect fetal growth.

## 1. Introduction

Caffeine, being a component of many popular products (tea and coffee), is widely consumed by pregnant women [1,2]. The half-life of caffeine is significantly prolonged in the body of a pregnant woman [3,4], due to decreased activity of the liver enzyme that is responsible for caffeine metabolism (by one-third in the first trimester of pregnancy and by half in the second trimester of pregnancy) [5]. The caffeine-induced increase in catecholamine concentrations (adrenaline, dopamine, and serotonin) interferes with placental blood flow and hampers transplacental nutrient transport to the fetus [6,7]. Caffeine and its metabolites easily cross the placental barrier [2,3,8], and caffeine excretion is delayed due to the immaturity of the fetal liver [2,9].

The impact of caffeine on the course of pregnancy and the development of the fetus is largely dependent on maternal intake and, supposedly, also on the speed of caffeine metabolism in the mother’s body [3,10]. Until recently, most experts believed that daily maternal intake of caffeine should not exceed 300 mg [9,10,11], although recent recommendations of the European Food Safety Authority (EFSA) and the American Institute of Medicine have limited the amount to 200 mg/day [12,13].

High maternal caffeine intake may lead to a miscarriage, premature birth, or low-birth neonatal weight but, despite extensive research, the evidence remains inconclusive [2,14]. The results of three meta-analyses, published between 2014 and 2016, of studies on caffeine intake and the risk for miscarriage seem to be the most unambiguous so far. According to these sources, a 100–150 mg increase in daily caffeine intake results in an elevated (by 7–19%) risk for miscarriage [14,15,16]. The risk increases by 40% among women who consume large amounts of caffeine (350–699 mg/day) as compared to small amounts (<50 mg) **[14]**. Nevertheless, research limitations of the abovementioned studies as far as methodology is concerned and lack of randomized trials, which yield the most credible results, need to be emphasized. As for premature birth, a meta-analysis of the available studies revealed no relationship between caffeine intake during pregnancy and the duration of pregnancy [11], nor has such a negative correlation been confirmed by a meta-analysis of studies on the risk for central nervous system defects in the fetus [17,18]. However, a relationship between maternal coffee intake and the risk for leukemia in the offspring has been suggested by meta-analyses of clinical case-control trials on the safety of coffee consumption [18,19].

The effects of maternal caffeine intake on the emotional development of their children remains yet another matter. While some authors found no evidence for the link between maternal caffeine intake (even over 300 mg/day) and the development of attention-deficit hyperactivity disorder (ADHD) in children aged 4–11 years [20,21,22], other researchers are less optimistic. A study from Denmark found that maternal consumption of ≥8 cups of coffee/day in the second trimester results in hyperexcitability in their children [23]. Noteworthy, caffeine citrate remains the gold standard in the treatment of apnea in premature newborns [24,25]. No adverse side effects have ever been reported [26], and some authors even observed a positive effect of such therapy on the psychomotor development of the affected children at the age of 18–22 months [27].

In light of a limited amount of data from Poland on caffeine intake during pregnancy, the aim of our study was to evaluate the level of maternal caffeine intake and its effect on neonatal anthropometric parameters.

## 2. Material and Methods

### 2.1. Study Design

The study was conducted among 100 pregnant women, who delivered at the Department of Obstetrics, Gynecology and Oncology, Medical University of Warsaw. The women presented at the hospital on weekdays (Monday–Friday), in the morning, during four months of 2014 and 2015. Approximately 20% of the women did not consent to participate in the study. The exclusion criteria were the following: non-Polish nationality, multiple gestation, advanced stage of the delivery, chronic maternal diseases before pregnancy, and threatened course of labor. A written informed consent was obtained from all participants. The local ethics committee approved of the study (no. 10/162/KB/2014). Maternal characteristics are presented in Table 1.

### 2.2. Data Collection

Caffeine intake from coffee and tea, which according to the available literature constitute the main sources of caffeine in the diet of pregnant women [1,3,22,28], were evaluated. Energy drinks were also included in the analysis, predominantly to investigate maternal attitudes to their consumption during pregnancy. Dietary caffeine intake from coffee and tea was investigated using a questionnaire, along with the type of coffee and the way of preparing infusions, since the brewing method is largely the factor behind caffeine content. Direct interviewing (face-to-face) was used and all interviews were conducted by the same dietitian (the main author of the manuscript) in order to ensure data homogeneity. The ‘Photo Album of Meals and Products’ was used to precisely evaluate portion size. Mean caffeine content values in coffee and tea brews were taken from our earlier analysis (Table 2) [28]. Neonatal data (sex, weight, length, Apgar score at 5 min., head and chest circumference) were obtained from the hospital medical records. The anthropometric measurements were taken by the midwives immediately upon delivery. Weight was measured using a physician beam scale. The remaining measurements were taken with the use of a tape measure. The total neonatal length was measured from the vertex of the head to the soles (with the feet kept vertical at 90 degrees). The occipital-frontal head circumference (tape was placed on the maximum protrusion of the occiput and supraorbital ridges) and the chest circumference (tape was placed horizontally on the sternum and lower tip of the shoulder blade) were measured.

### 2.3. Statistical Analysis

The normal distribution of all studied parameters was checked using the Kolmogorov–Smirnov test. The Mann–Whitney test was used to compare the distribution of caffeine intake between independent groups (education, age, place of residence, smoking, gestational diabetes, and pregnancy-induced hypertension). A multivariate logistic regression model was used to investigate a relationship between caffeine intake and other factors (calcium intake, use of dietary supplements, pre-pregnancy body mass index (BMI), weight gain during pregnancy, smoking, gestational diabetes, maternal age and education, gravidity, professional activity during pregnancy, and sex of the neonate) versus neonatal weight, length, head and chest circumference lower than the median. Only term deliveries (94 newborns) were included into the analysis. Using the method of step elimination with 0.1 level for staying in the model, statistically significant factors were selected at a significance level of 5%. The relation of statistically significant factors was expressed by the odds ratio (OR) and the 95% confidence interval (95% CI).

## 3. Results

### 3.1. Caffeine Intake

Mean caffeine intake among the pregnant women from our study was 68 ± 51 mg/day. A vast majority of the women (79%) consumed <100 mg of caffeine, while the remaining 19% and 2% of the respondents consumed 100–200 mg and >200 mg/day, respectively. None of the subjects exceeded the dose of 300 mg of caffeine/day.

Tea was the source of 63% (43 mg) of total caffeine, and the remaining 37% came from coffee. Only 2 (2%) out of all respondents declared sporadic use of energy drinks, and for this reason these products were not included in evaluation of total caffeine intake.

Black tea supplied 4-fold more caffeine than green tea (34 ± 33 mg and 9 ± 26 mg, respectively). No statistically significant differences were found between caffeine intake and maternal age, education, place of inhabitance, smoking, gestational diabetes mellitus, or pregnancy-induced hypertension.

### 3.2. Caffeine Exposure and Neonatal Anthropometric Parameters

Maternal caffeine intake was not linked with neonatal anthropometric parameters (weight, length, head and chest circumference) (*p* > 0.05). Neonatal characteristics are presented in Table 1. Maternal weight gain during pregnancy was the parameter that turned out to be related to neonatal length. Pregnant women with too low weight gain are at a 3-fold higher risk for giving birth to infants with lower than median length for term neonates as compared to women with either recommended or excessive weight gain (Table 3).

### 3.3. Coffee, Tea, and Energy Drinks Consumption

Tea and/or coffee brews were very popular in the diet of pregnant women. Only 10% of the respondents declared complete abstinence. Coffee was consumed by 43% of the women, including 1 subject who consumed only decaffeinated coffee. Instant coffee was the most popular drink (31%), and only 2% of the respondents consumed coffee from a coffee maker (Table 4). Daily consumption of coffee was declared by 32% of the women, mostly 1 cup/day (26%), and only 1 subject drank 3 cups of coffee/day. Mean coffee consumption in the entire study population was 74 ± 117 mL/day. All women consumed light coffee brews (i.e., 1 teaspoon of coffee per cup).

Tea consumption was reported by 80% of the respondents, including 72% who consumed tea every day, while the remaining women drank tea several times a week or less (Table 4). The amount of tea consumption varied between 2 cups (26%), 1 cup (21%), 3 cups (15%), or 4–8 cups (10%) a day. Mean tea consumption in the entire study population was 346 ± 379 mL/day. The vast majority of the women consumed only black tea (60%), mainly tea bags (90% of tea drinkers), whereas only 10% used tea leaves. As for brew strength, 84% of the tea bag drinkers declared that they preferred light- or medium-intensity brews (up to 1 min), and only 16% brewed the tea longer.

## 4. Discussion

In our study, we detected a small caffeine intake among the investigated population, significantly below 100 mg/day. Bearing in mind that, according to the literature, coffee and tea are the main sources of that component in the diet of pregnant women (80–90%) [1,22], it seems possible to conclude that the amount consumed is at a safe level, even taking into account consumption of other products with caffeine content.

To the best of our knowledge, only two studies on caffeine intake during pregnancy have been conducted in Poland so far, and both report optimistic findings. Mean daily caffeine intake was 91 mg/day according to the first study (conducted between 2005–2007) and 50 mg/day according to the second study (conducted between 2014–2015) [28,29]. The current result (68 mg from coffee, tea, and energy drinks) confirmed that consumption of caffeinated products by women in Poland during pregnancy is reasonable and non-excessive. Also, other data revealed that 73% of the Polish pregnant women declared an awareness of the potentially negative impact of coffee on the developing fetus [30]. Until recently, the amount of over 300 mg of caffeine/day was considered excessive and such consumption was reported for 1.6% of the investigated women [28]. Lately however, the so-called ‘safe’ dose of caffeine was significantly lowered (to 200 mg), but still only 2% of our study population and 1.4% of the subjects in the study of Błaszczyk-Bębenek et al. [29] exceeded the recommended dose. Mean caffeine intake among pregnant women in the US, Great Britain, and Sweden has been estimated at 58–125 mg, 159 mg, and 215 mg per day, respectively [9,31,32]. Very high (mean 258 mg/day) caffeine intake was observed in Japan, where over 67% of pregnant women consume over 200 mg/day [3]. In contrast, a surprisingly low (median 44–62 mg/day) caffeine intake among pregnant women was reported in Norway [1], whose inhabitants are well-known coffee lovers [2].

A relatively low caffeine intake in our study may be the result of a decision to reduce coffee consumption during pregnancy. In studies by Jarosz et al. [28], and by Wyka et al. [30], 26% and 19% of the study population, respectively, chose not to drink coffee during pregnancy. Similar findings have been reported by authors from other countries, where reduced tea and coffee consumption was the most common modification in the diet of pregnant women [33]. In our study, 43% of the respondents declared coffee consumption, which is consistent with the national data (39–52% of women) [28,30,34]. Espresso, which contains more caffeine than other coffee brews [1,35], has seldom been consumed by pregnant women in Poland, which might also account for the low caffeine intake we detected. Tea, whose consumption was declared by 80% of the respondents in this study and 93% in another study, is decidedly more popular and continues to be the main source of caffeine in the diet of pregnant women from Poland [28]. In Poland, black tea is the most popular drink and the main source of daily caffeine intake (44–59% according to the earlier studies [28,29] and 50% according to the current study), and only a small amount is derived from green tea (5–16% according to the earlier studies [28,29] and 13% according to the current study). Tea is also the main source of caffeine in Great Britain [9] and Japan [22], although in Japan, most caffeine in the diet of pregnant women comes from green tea (75%), and only some from black tea (4%). In contrast, coffee remains the main caffeine source in the Scandinavian countries, the US, and Canada [1,3,28,36]. In our study, we found that pregnant women avoid energy drinks, which is consistent with reports from Western European countries, where only 1–2% of total caffeine content in the diet of pregnant women is supplied by energy drinks [9,12,28].

The results of the Care Study Group from Great Britain were the reason why EFSA lowered the safety threshold (to 200 mg) for daily intake of caffeine during pregnancy. The study revealed that caffeine intake over 200 mg/day results in a 60–70 g decrease in neonatal weight [9]. In our study, we found no relationship between neonatal anthropometric parameters and caffeine intake. Importantly, mean caffeine intake was significantly below the permissible dose (i.e., 200 mg/day). No relationship between neonatal anthropomorphic parameters and caffeine intake in Poland was found in our previous study as well, where mean caffeine intake was <100 mg, which is similar to the findings in the present study [28]. According to the latest reports in the literature, in particular a study from Norway, daily caffeine intake of <200 mg increases the risk for small-for-gestational-age infant by 16% [37]. In a study from Ireland, a daily increase in caffeine intake by 100 mg resulted in a decrease in neonatal weight (by 72 g), length (by 0.3 cm), and head circumference (by 0.12 cm) [38]. On the other hand, a study from Brazil revealed no relationship between high caffeine intake (≥300 mg) and low-birth-weight (LBW) neonates [39]. In light of the recent meta-analyses, Rhee et al. in their meta-analysis of eight cohort and four case-control studies concluded that high maternal intake of caffeine increases the risk for LBW neonate by 38% [7], while Greenwood et al., in their meta-analysis of 26 cohort and 27 case-control studies, found that increased caffeine intake (by 100 mg) results in higher risk (by 7%) for LBW neonate [15]. Some experts are of the opinion that neonates born to non-smoking mothers who consume ≥300 mg of caffeine/day, but only those who metabolize caffeine fast (i.e., AA genotype), are at higher risk for delivering infants with decreased birth size [40].

Several limitations of the present study might have biased the final results, chief among them a small sample size, which was the result of the number of deliveries at the clinic, but also the fact that it was a pilot study. It was a preliminary study to recognize the attitudes of pregnant women to coffee consumption after the introduction of a coffee cup into the graphic representation of the nutrition guidelines (food pyramid) in Poland. Also, we collected data on maternal caffeine intake on the day of the delivery, so the study was retrospective in nature. Nonetheless, drinking coffee and tea is a common component of many individuals’ eating habits and it should not be problematic to recall the frequency of their consumption, even from the time perspective. Also, caffeine intake might have been different throughout the pregnancy, although various studies reported lack of significant differences between caffeine intake and pregnancy trimesters [9,39]. Furthermore, the questionnaire did not include information about other sources of caffeine, such as soft drinks, but many authors have previously reported that coffee and tea are the sources of over 80% of the caffeine in the diets of pregnant women [1,22,38]. Our data included information on types of coffee (e.g., instant, brewed), as well as the intensity of tea and coffee brews, which to a large extent is the decisive factor for determining caffeine content in a drink and allows for a precise evaluation of the intake.

## 5. Conclusions

Caffeine intake among our study population was relatively low, which resulted from low coffee consumption. Tea, due to its higher popularity during pregnancy, constituted the main source of caffeine. No relationship was found between such caffeine intake and neonatal anthropometric parameters.

## Figures and Tables

**Table 1:** Maternal and neonatal characteristics.

Maternal Characteristics	
**Number of Women**	100
age (in years) mean ± SD	30.0 ± 4.4
education higher (%) other (%)	66 34
place of residence Warsaw (%) other (%)	58 42
parity primipara (%) multipara (%)	42 58
premature birth (%)	6
pre-pregnancy BMI (mean) ± SD	22.7 ± 3.8
gestational diabetes (%)	11
pregnancy-induced hypertension (%)	9
smoking during pregnancy (%)	15
professionally active during pregnancy (%)	58
daily calcium consumption—from milk and dairy products (mg)median (min–max)	598 (69–1872)
supplementation with vitamin/mineral preparations (%)	89
**Neonatal** **Characteristics**	
number of newborns	94
gestational age (weeks) mean ± SD	39.4 ± 1.0
neonatal weight (g) median (min–max)	3530 (2390–4650)
LBW neonates (<2500 g), *n* (%)	1 (1.1)
macrosomia (>4000 g), *n* (%)	19 (20.2)
neonatal length (cm) median (min–max)	56 (50–60)
neonatal head circumference (cm) median (min–max)	35 (32.5–38.0)
neonatal chest circumference (cm) median (min–max)	34 (29–38)
Apgar score (points) mean ± SD	9.9 ± 0.1

**Table 2:** Caffeine content in coffee and tea brews used to evaluate caffeine intake by the pregnant women.

Product	Portion Size (mL)	Caffeine Content (mg)
brewed coffee (boiling water poured over ground coffee in a cup): 1-teaspoon brew 2-teaspoon brew	160 160	36 74
instant coffee: 1-teaspoon brew 2-teaspoon brew	160 160	61 117
black tea: 1-min brew 5-min brew	200 200	22 33
green tea: 1-min brew 5-min brew	200 200	22 33

**Table 3:** Analysis of the influence of maternal caffeine intake and other factors on the risk for neonatal length below the median.

*N* = 94	OR (95% CI)	*p*-Value
Caffeine intake: >100 mg/day vs. ≤100 mg/day	2.52 (0.86; 7.40)	0.092
Calcium intake: >611 mg/day vs. ≤611 mg/day		>0.1
Supplementation with vitamin/mineral preparations		>0.1
Pre-pregnancy BMI: underweight vs. normal overweight/obesity vs. normal		>0.1
Gestational weight gain: too low vs. recommended and excessive	3.01 (1.08; 8.3)	0.034
Smoking		>0.1
Gestational diabetes		>0.1
Age (years): >30 vs. ≤30		>0.1
Education: secondary vs. higher	0.38 (0.15; 1.00)	0.051
Gravidity: primiparas vs. multiparas		>0.1
Professional activity during pregnancy		>0.1
Neonatal sex		>0.1

BMI: body mass index.

**Table 4:** Coffee and tea consumption among pregnant women.

	Number of Women (%)
**Coffee**	43
instant	31
brewed in a cup in a coffee maker	12 10 2
**Consumption Frequency**	
every day	32
3–4 times a week	4
1–2 times a week	8
2–3 times a month	1
**Tea**	80
black	60
green	6
black and green	14
**Consumption Frequency**	
every day	72
3–4 times a week	5
1–2 times a week	2
2–3 times a month	1
